# Docetaxel-loaded M1 macrophage-derived exosomes for a safe and efficient chemoimmunotherapy of breast cancer

**DOI:** 10.1186/s12951-022-01526-2

**Published:** 2022-08-02

**Authors:** Yongmei Zhao, Yuanlin Zheng, Yan Zhu, Hongyun Li, Hongyan Zhu, Tianqing Liu

**Affiliations:** 1 School of Pharmacy, Nantong University, Nantong, 226001 China; 2 NICM Health Research Institute, Western Sydney University, Westmead, NSW 2145 Australia

**Keywords:** Exosomes, Macrophage polarization, Tumor immunity, Mitochondrial functions, Breast cancer

## Abstract

The conversion of tumor-promoting M2 macrophage phenotype to tumor-suppressing M1 macrophages is a promising therapeutic approach for cancer treatment. However, the tumor normally provides an abundance of M2 macrophage stimuli, which creates an M2 macrophage-dominant immunosuppressive microenvironment. In our study, docetaxel (DTX) as chemotherapeutic modularity was loaded into M1 macrophage-derived exosomes (M1-Exo) with M1 proinflammatory nature to establish DTX-M1-Exo drug delivery system. We found that DTX-M1-Exo induced naïve M0 macrophages to polarize to M1 phenotype, while failed to repolarize to M2 macrophages upon Interleukin 4 restimulation due to impaired mitochondrial function. This suggests that DTX-M1-Exo can achieve long-term robust M1 activation in immunosuppressive tumor microenvironment. The in vivo results further confirmed that DTX-M1-Exo has a beneficial effect on macrophage infiltration and activation in the tumor tissues. Thus, DTX-M1-Exo is a novel macrophage polarization strategy via combined chemotherapy and immunotherapy to achieve great antitumor therapeutic efficacy.

**Supplementary Information:**

The online version contains supplementary material available at 10.1186/s12951-022-01526-2.

## Introduction

Tumor-associated macrophages (TAMs) are essential components of the immune responses in tumor microenvironment and play an important role in tumor growth, metastasis, and drug therapy [1, 2]. They can be recruited to the tumor site to revoke and evoke tumoricidal activity via altering their functional plasticity. The two activation polarization phenotypes include pro-inflammatory (M1/M1-like) and anti-inflammatory (M2/M2-like). M1 macrophages can be stimulated by pro-inflammatory cytokines like interferon-γ (IFN-γ) or lipopolysaccharides (LPS) to produce high level of proinflammatory and immunostimulatory cytokines, leading to tumor suppression [3]. In contrast, activated M2 macrophages release immunosuppressive cytokines to promote tumor progression and metastasis [4, 5]. Therefore, reprogramming of macrophage polarization from tumor-promoting M2 to tumor-suppressing M1 macrophages is a promising therapeutic approach for cancer treatment [6–8]. However, tumor microenvironment normally facilitate the functionality of TAMs toward a tumor-promoting M2 phenotype [9, 10]. Although efforts have been made to induce this phenotypical switch by using pro-inflammatory cytokines, antibodies or nanoparticles [7, 11–13], the main challenge is to maintain high potency of M1 polarization in the highly dynamic tumor microenvironment. The reduced tumoricidal macrophage performance can lead to less reliable cancer immunotherapy. Hence, there is an urgent need to develop novel immunotherapy strategies based on long-lasting high potent M1 macrophages.

Mitochondria, as the center of cellular metabolism, are critical for energy supply, biosynthesis and redox balance. Growing evidences have demonstrated that mitochondrial metabolism is responsible for macrophage polarization and function alteration. It is reported that metabolic manipulation of M1 cells via inhibition of mitochondrial function can lead to irreversibility of M1 to M2 polarization [14]. More recently, Gu et al. designed biocompatible iron-based metal organic framework nanoparticles with a ferroptosis activator. This system disrupted mitochondrial functions and induced the energy metabolism of macrophages from OXPHO to glycolysis via enhanced ferroptosis-associated stress, thus leading to potent tumoricidal activities of M1 macrophages [15]. These findings suggest that mitochondria-targeted immunometabolism programming have a great potential as a therapeutic strategy to trigger long-term M1 activation for cancer treatment.

Interestingly, certain chemotherapeutic drugs can also activate cytotoxic immune responses against tumor cells. For example, docetaxel (DTX) was able to deplete immunosuppressive M2 macrophages while activate tumoricidal M1 macrophages in 4T1-Neu mammary tumor implants [16]. Although the chemotherapy-based immunotherapy is emerging as a promising therapeutic approach, the mechanism of action governing the macrophage activation is not completely understood. Herein, for the first time, we report chemotherapy-enabled macrophage programming strategy via modulating mitochondrial functions, aiming to achieve highly effective immunotherapy. Taking advantages of the proinflammatory nature and drug delivery properties of M1 macrophage-derived exosomes (M1-Exo) [17], we established DTX-loaded M1-Exo drug delivery system (DTX-M1-Exo) to reactivate tumor immuno-microenvironment for breast cancer treatment. We observed that DTX-M1-Exo induced naïve M0 macrophages to polarize to M1 activation, while failed to repolarize to M2 upon Interleukin 4 (IL-4) restimulation due to impaired mitochondrial function. The in vivo results confirmed that DTX-M1-Exo played a positive role in macrophage infiltration and activation of the high potent M1 macrophage population in the tumor region, leading to significant tumor inhibition. We provided evidence that DTX-M1-Exo, as a combined chemotherapy and immunotherapy, had excellent antitumor therapeutic efficacy via macrophage polarization with high potency.

## Method

### Cell culture and macrophage polarization

Murine carcinoma cancer cell line 4T1 and murine macrophage cell line RAW264.7 were purchased from American Type Tissue Collection (ATCC) and maintained in Dulbecco’s modified Eagle’s medium (DMEM; Gibco, Thermo Fisher Scientific) supplemented with 10% feral bovine serum (FBS; Thermo Fisher Scientific) and 1% penicillin/streptomycin (PS, Thermo Fisher Scientific) in a humidified incubator at 37 °C with 5% CO_2_. Exosomes-free FBS were prepared by ultracentrifugation at 120,000*g* at 4 °C for 16 h. Cells were routinely tested and mycoplasma-free.

When RAW264.7 cells (naïve M0 macrophages) reached about 60% confluence, they were stimulated with 100 ng/mL LPS (Sigma-Aldrich) and 20 ng/mL IFN-γ (Sigma-Aldrich) for 24 h to induce M1 macrophage polarization. RAW264.7 cells were treated with 20 ng/mL of IL-4 (Sigma-Aldrich) to induce M2 macrophage polarization.

### Exosome isolation and characterization

To obtain M0-Exo or M1-Exo, the culture media of M0 macrophages or activated M1 macrophages were collected and centrifugated at 800×*g*, 3000×*g*, and 10,000×*g* for 10 min, 10 min, and 30 min, respectively, at 4 °C, to remove cell debris and large extracellular vesicles. Then the culture media were ultra-centrifugated at 100,000*g* with a type Ti41 rotor (Optima XPN-100, Beckman Coulter) for 70 min at 4 °C to obtain the M0-Exo or M1-Exo. The weight of exosomes was determined by a well-established quantification method of total protein using a Micro BCA Protein Assay kit. After constructing a protein standard curve (0, 0.025, 0.05, 0.1, 0.2, 0.3, 0.4 and 0.5 μg/μL) by measuring absorbance at 562 nm, the corresponding protein concentrations of different experimental groups were calculated.

The morphology of both M0-Exo and M1-Exo were characterized using a transmission electron microscope (TEM, Talos F200X). The isolated exosomes were diluted in PBS and 10 μL of M0-Exo or M1-Exo solution were dropped onto carbon-coated copper grids (Sigma-Aldrich). After drying for 5 min, 1% uranium acetate (Sigma-Aldrich) was added to stain the samples for 1 min. Samples were dried for another 20 min and examined by TEM. Hydrodynamic particle size was measured by dynamic light scattering (DLS, Nano-zs30, Malvern).

### Drug loading and quantification

DTX (Sigma-Aldrich) were loaded into either M0-Exo or M1-Exo using a standard electroporation method. In brief, DTX and exosomes were mixed in 400 µL of PBS solution at a weight ratio of 6:1. The mixture was added into 4 mm path length electroporation cuvettes and electroporated under optimal condition of 400 V for voltage and 150 mF for electric capacity with 1 ms discharging time using a Bio-Rad electroporation instrument. The exosomal membrane was recovered after incubated at 37 °C for 30 min. The suspension was then centrifuged at 100,000*g* for 60 min and filtered with an amicon filter (100 kDa, Merk Millipore), to remove extra exosomal DTX. The DTX-M0-Exo and DTX-M1-Exo were collected and stored at 4 °C for the following experiments.

The amount of drug loading in the exosomes was measured by dissolving DTX-M0-Exo or DTX-M1-Exo with methanol to completely release DTX. Then the DTX content was quantified by measuring by a high performance liquid chromatography with ultraviolet (HPLC–UV) equipped with a Waters C18 column (Knauer) at a wavelength of 230 nm.

### In vitro cellular uptake

To investigate the intracellular uptake of M0-Exo and M1-Exo, 4T1 cells were seeded on Thermanox™ coverslips in a 6-well plate and cultured with cell culture media overnight. The cells were then treated with M0-Exo and M1-Exo labelled with 20 μg/mL of green fluorescent dye PKH67 (Sigma-Aldrich) for 6 h. After treatment, the cells were fixed with 4% paraformaldehyde, permeabilized with 0.3% Triton X-100, and counter-stained by nuclei dye DAPI (Sigma-Aldrich) for 10 min. The prepared samples were imaged using a confocal laser scanning microscope (CLSM, Zeiss 780-NLO).

### In vitro cell proliferation

To evaluate the in vitro anti-cancer effects of DTX-loaded exosomes on the 4T1 cells, cells were seeded into 96-well plates (1000 cells/well, 3 wells/group) and cultured for 6 h before treatment. The cells were then treated with control, DTX, M0-Exo, M1-Exo, DTX-M0-Exo, and DTX-M1-Exo in cell culture media for up to 144 h. During treatment, each group was measured cell growth every 3 h using the IncuCyte ZOOM Live-Cell Analysis System (Essen BioScience). Data were analyzed using the IncuCyte software.

### Real-time quantitative polymerase chain reaction (qPCR) analysis

The cells were harvested and tested by real-time qPCR assays. The total RNA in each sample was extracted by TRIzol (Thermo Fisher Scientific) as per the manufacturer’s instructions. After confirmed the RNA quality by formaldehyde gel electrophoresis and UV spectroscopy, complementary DNA (cDNA) was synthesized using Superscript III Reverse Transcriptase (ThermoFisher Scientific) and primers as per the manufacturer’s instructions. Real-time qPCR was performed on a Light Cycler 480 (Roche Diagnostics). The primers and cycling conditions were described previously [15, 18].

**Table:** 

Primer	Forward (5’-3’)	Reverse (5’-3’)
Arg-1	TACAAGACAGGGCTCCTTTCAG	CGTTGAGTTCCGAAGCAAGC
CD206	GCTGGCGAGCATCAAGAGTA	AGGAAACGGGAGAACCATCAC
iNOS	TGCTTTGTGCGAAGTGTCAG	CCCTTTGTGCTGGGAGTCAT
TNF-α	GCCTATGTCTCAGCCTCTTCTC	GCCATTTGGGAACTTCTCATCC

### Determination of mitochondrial membrane potential and mitochondrial oxidative stress

To test the mitochondrial functions, the naïve M0 macrophages were treated with PBS control, LPS + IFN-γ, IL-4, DTX, M0-Exo, M1-Exo, DTX-M0-Exo, and DTX-M1-Exo. Mitochondrial membrane potentials of treated macrophages were stained with mitochondrial membrane potential dye tetramethylrhodamine methyl ester (TMRM, Sigma-Aldrich) and measured by flow cytometry (FACS CANTO II, BD Biosciences). Mitochondrial reactive oxygen species (ROS) of the treated macrophages was stained with MitoSOX dye (Thermo Fisher Scientific) at the concentration of 5 µM and incubated for 20 min in the dark and analyzed by flow cytometry.

### In vivo antitumor efficacy study

All animal experiments were approved by the Animal Ethics Committee of Nantong University. BALB/C female mice (4–6 weeks) were ordered from the Laboratory Animal Center of Nantong University and were randomly assigned to four groups of 5 mice per group. 4T1 cells (5 × 10^5^ cells) were subcutaneously injected into right flanks of Balb/C mice. Tumor growth was monitored daily and measured using digital calipers. Tumor volume was calculated using the following formula: tumor volume = (length [mm])^2^  ×  (height [mm])  ×  π / 6. When the size of tumor reached 60 mm^3^, mice were treated with saline control, DTX, M1-Exo, and DTX-M1-Exo. Drug treatment was administered intravenously at a corresponding dose of 5 mg/kg DTX every 3 days for a total of four doses, initiated on Day 1.

### Immunofluorescent staining

Tumor tissues were harvested, fixed by 4% paraformaldehyde and embedded in paraffin. The embedded samples were sectioned into 5 μm thick histologic slides and blocked with blocking buffer. Primary antibody against F4/80 (1:2000, Rat anti-mouse monoclonal antibody, Abcam) were applied onto the sections for 1 h at room temperature. After washing, they were incubated with Alexa Fluor 594-labelled secondary antibody (Thermo Fisher Scientific) for 1 h. Primary antibodies against M1 marker CD86 (1:2000, Rabbit anti-mouse monoclonal antibody, Affinity) and M2 marker CD163 (1:2000, Rabbit anti-mouse monoclonal antibody, Affinity) was also applied onto the sections for 2 h at room temperature. After washing, they were incubated with Alexa Fluor 546-labelled secondary antibody (Life Technologies) for 1 h at room temperature. The sections were washed and mounted using the ProLong Gold Antifade Mountant with DAPI. The slides were visualized using a confocal laser scanning microscopy.

### Immunohistochemistry

The paraffin-embedded sections were stained with hematoxylin and eosin (H&E) using a Leica Autostainer XL (ST5015, Leica Microsytem) for tissue histological analysis. Expression of Ki67 was investigated by immunohistochemical analysis using anti-Ki67 primary antibody (Cell Signalling) and an HRP-conjugated anti-rabbit secondary antibody (Jackson Immunresearch) following DAB enhancement (Sigma). The prepared specimens were imaged with a brightfield microscope. Sections without primary antibodies were used as negative controls and showed no immunoreactivity.

### Statistical analysis

All experimental data were obtained in triplicate unless otherwise mentioned. Results were presented as mean ± standard deviations and statistical significance was examined by an unpaired two-tailed Student’s t-test by the GraphPad Prism 9.0 software. The *P*-value < 0.05 was considered as statistically significant.

## Result and discussion

### M1-Exo preparation, characterization and docetaxel loading

**Fig. 1:**
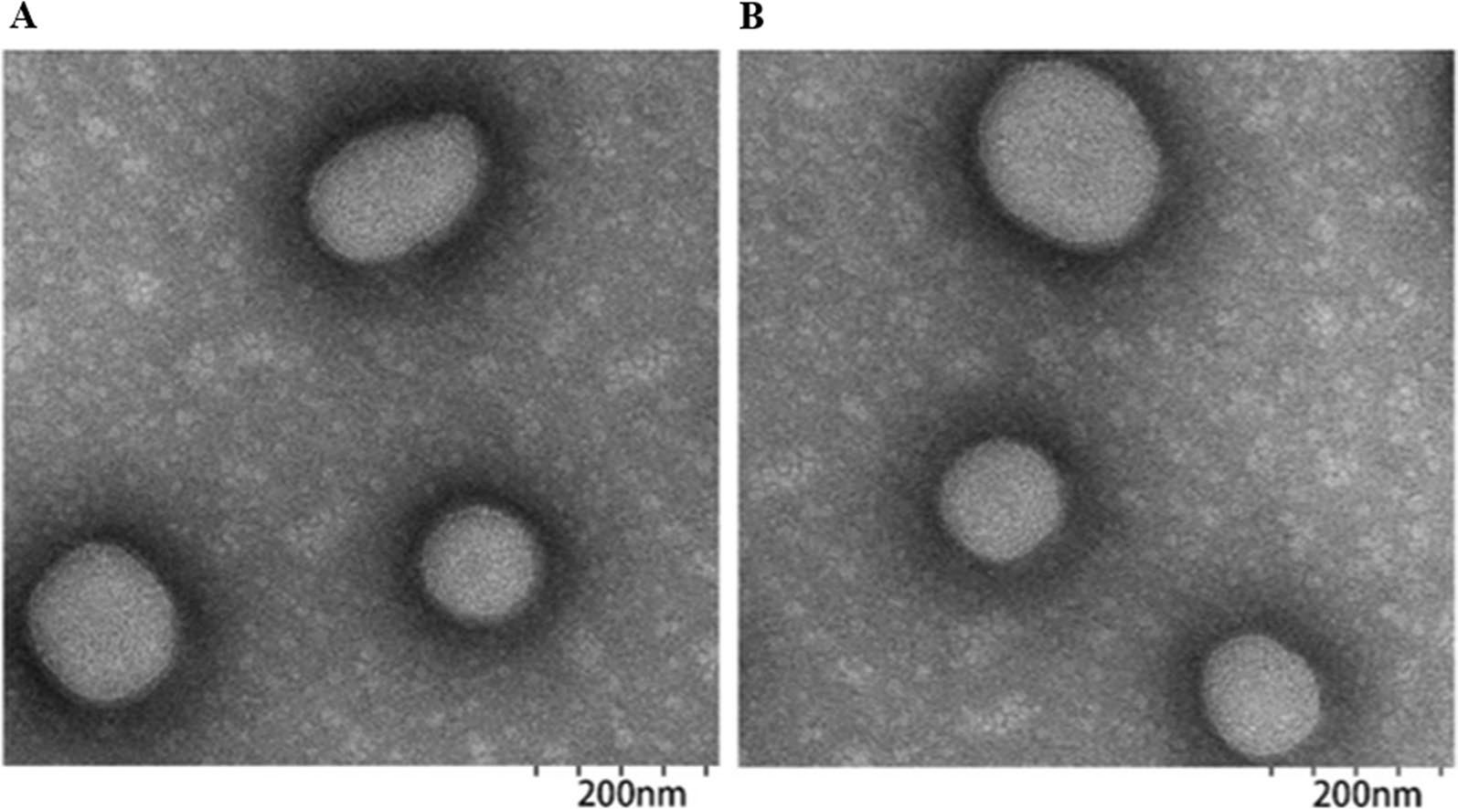
Morphological characterization of exosomes with or without drug loading: TEM images of (**A**) M1-Exo and (**B**) DTX-M1-Exo

In order to induce the polarization of macrophages into M1, naïve macrophages were incubated with LPS and IFN-γ for 24 h. Exosome was then obtained based on a well-established differential centrifugation method [19, 20]. The morphology of M1-Exo and DTX-M1-Exo was further confirmed by TEM and showed that they possess well dispersed, spherical shape with uniform diameter (Fig. 1A and B). The hydrodynamic size of the isolated M1-Exo, M0-Exo and drug loaded DTX-M0-Exo, DTX-M1-Exo nanoparticles were measured by DLS which showed a relatively average size around 105.2 ± 0.8, 101.2 ± 0.3, 151.8 ± 0.6 and 163.5 ± 0.4 nm in diameter, respectively (Table 1). These results suggested that the sizes of exosomes were slightly increased after drug loading. By using electroporation method, both of M0-Exo and M1-Exo showed high drug loading efficiency that around 15.57 ± 1.7% and 17.62 ± 2.58% of DTX was achieved, respectively (Table 1).

**Table 1:** Physical properties and drug loading efficiency of exosome formulations

Sample	Hydrodynamic sze D_h_^a^ (nm)	Zeta-potential (mV)	Drug loading (%)
M0-Exo	105.2 ± 0.8	35.45 ± 1.72	–
M1-Exo	101.2 ± 0.3	36.81 ± 2.43	–
DTX-M0-Exo	151.8 ± 0.6	30.76 ± 1.21	15.57 ± 1.7
DTX-M1-Exo	163.5 ± 0.4	32.33 ± 3.63	17.62 ± 2.58

^a^Determined by DLS

### In vitro cellular uptake and cell viability

**Fig. 2:**
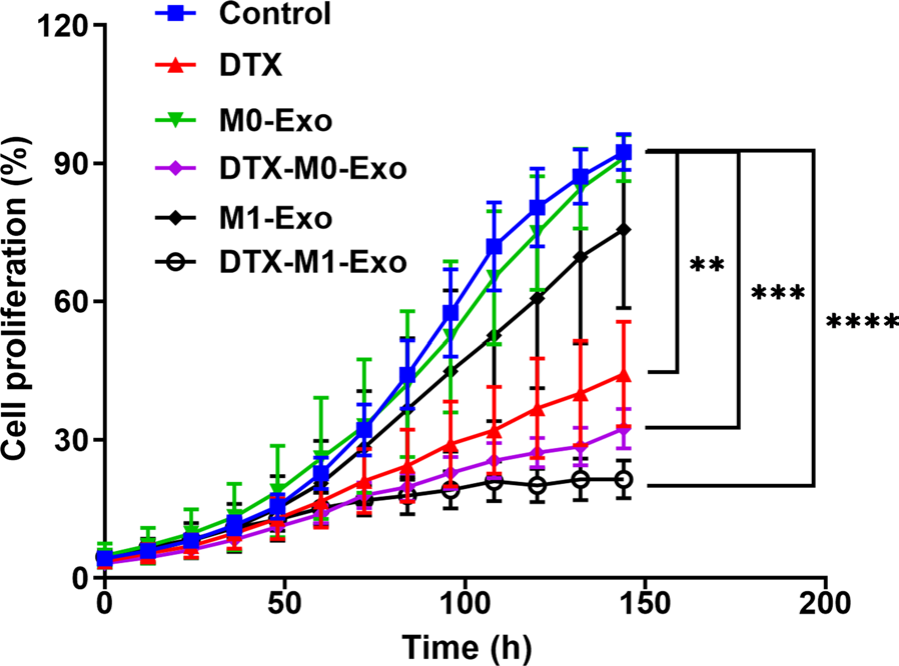
Cell cytotoxicity study of control, DTX, M0-Exo, DTX-M0-Exo, M1-Exo, and DTX-M1-Exo against 4T1 breast cancer cells. (** *P* < 0.01, *** *P* < 0.001, **** *P* < 0.0001 vs control)

Confocal laser scanning microscopy (CLSM) was used to image the cellular uptake of exosomes as drug vesicles. M1-Exo and M0-Exo, loaded with green fluorescent dye PKH67, was incubated with 4T1 cancer cells for 6 h, separately. As shown in Fig. 2, M1-Exo and M0-Exo were efficiently internalized by the cells. As they are over 100 nm in size, they are more likely to be taken up via endocytic and phagocytotic pathways. M1-Exo showed increased uptake efficiency compared with M0-Exo, which may be attributed to the specific recognition of the surface markers. The high cellular internalization ability of M1-Exo is consistent with other studies showing the phospholipid bilayers of exosomes directly fuse with cell plasma membrane and release the cargo/content into the cytosol of target cells [21]. Taking advantage of this fusion and release behavior, they can improve chemotherapeutic drug delivery for cancer treatment.

**Fig. 3:**
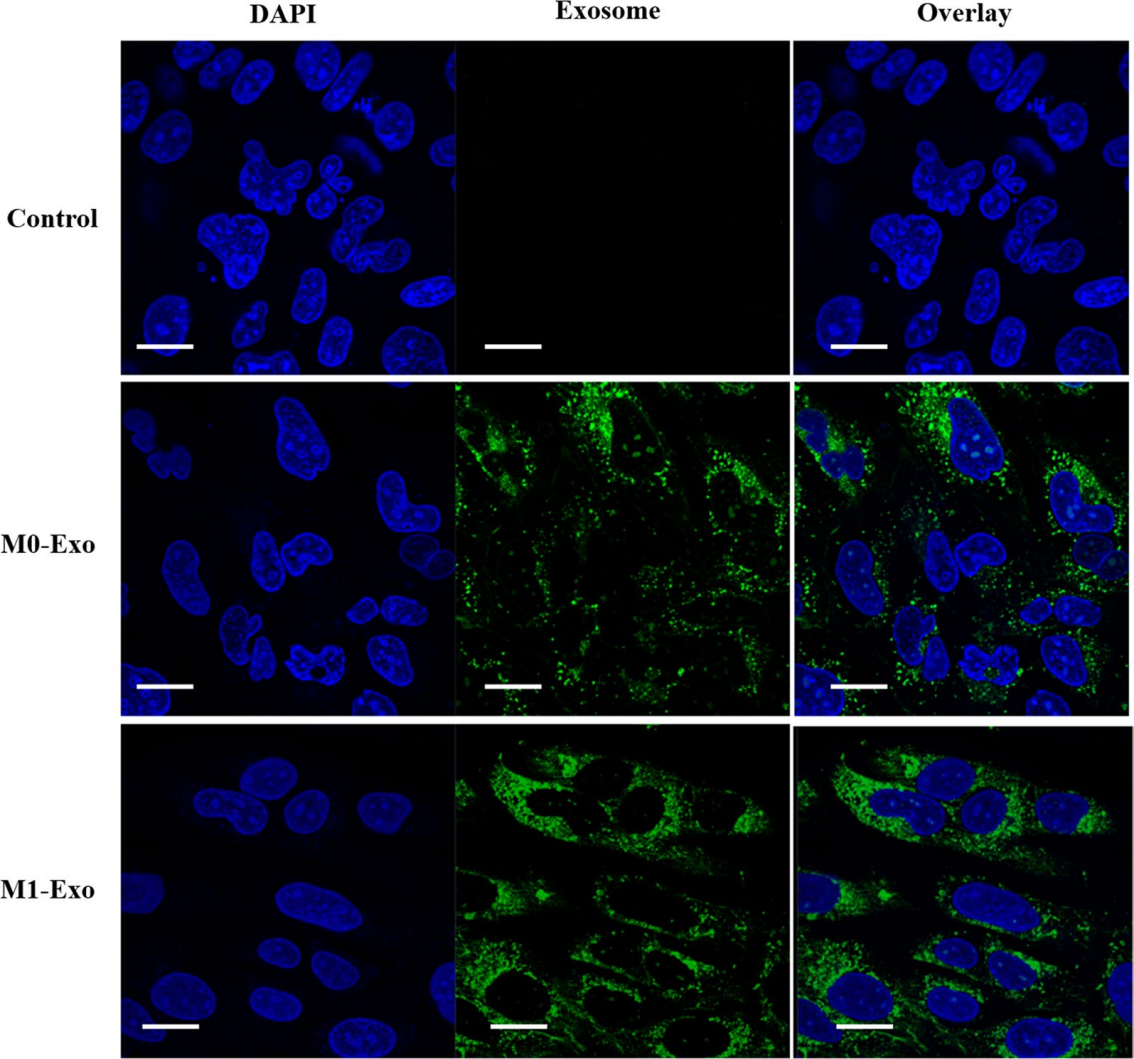
The cellular uptake behavior of the exosomal vesicles (M1-Exo or M1-Exo) toward 4T1 cancer cells after 6 h incubation. In the CLSM images, cell nuclei were stained by DAPI (blue), while the exosomes were stained by fluorescent dye PKH67 (green). The scale bar is 20 µm

To investigate in vitro therapeutic efficacy, cell proliferation study was carried out on 4T1 breast cancer cells treated with DTX, M0-Exo, DTX-M0-Exo, M1-Exo, and DTX-M1-Exo. The cytotoxicity data is summarized in Fig. 3 at the same concentration of DXT. M0-Exo exhibited negligible effects on 4T1 cells, while cells treated with M1-Exo slightly inhibited cellular growth when compared to M0-Exo, suggesting that M1-Exo themselves benefit cancer treatment. DTX-M1-Exo significantly reduced cancer cell proliferation and induced greatest cytotoxicity to the cancer cells over the treatment period compared to other groups. This indicated that DTX-M1-Exo efficiently improved the anticancer efficiency. The cancer-killing capacity of DTX-M1-Exo correlated with the high cellular uptake ability of M1-Exo as observed previously. DTX-M0-Exo also had slightly better anti-proliferative effects on 4T1 cells compared to DTX alone, which may be attributed to better cellular internalization ability of exosomes too [21].

### DTX-M1-Exo induced naïve macrophage differentiation to M1 phenotype

**Fig. 4:**
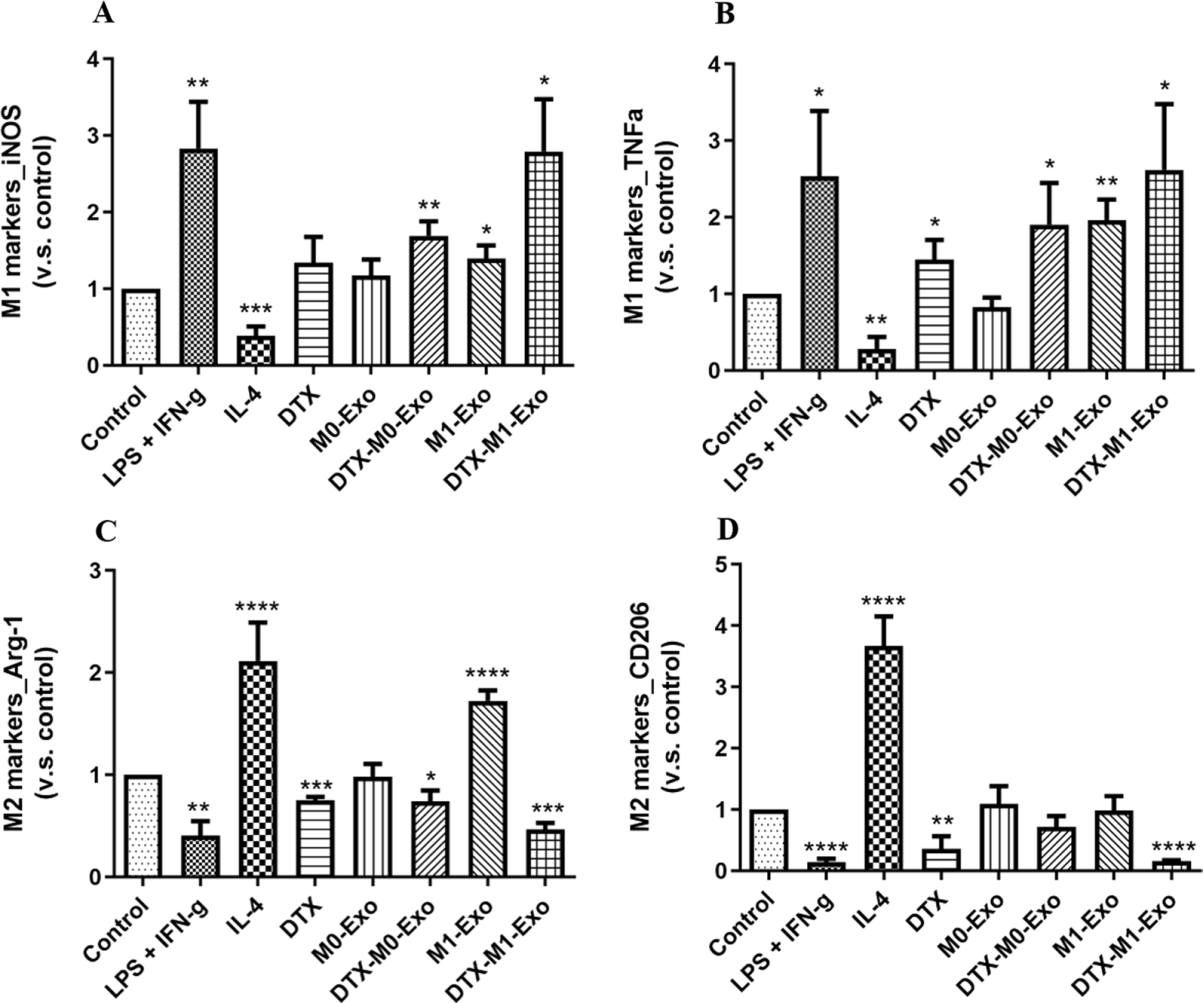
Macrophage phenotypes after treatment of IL-4, LPS + IFN-γ, DTX, M0-Exo, DTX-M0-Exo, M0-Exo, and DTX-M1-Exo, by measuring the expression of M1-macrophage markers (**A**) *iNOS* and (**B**) *TNF-α*, as well as M2-macrophage markers (**C**) *Arg-1* and (**D**) *CD206*. (* *P* < 0.05, ** *P* < 0.01, *** *P* < 0.001, **** *P* < 0.0001 vs. the control group)

To assess the effects of DTX-M1-Exo on macrophage polarization, the gene expression of M1 and M2 associated markers of treated macrophage cells were analyzed. As expected, LPS+IFN-γ stimulation induced M1 polarization by increasing the expression of M1-specific markers *iNOS* and *TNF-α* and reducing the expression of M2-specific markers *Arg-1* and *CD206*, while IL-4 stimulation promoted M2 polarization, confirmed by the gene expression (Fig. 4). This confirmed that the standard macrophage polarization was established to be used as controls for further comparison. M1-Exo treated macrophages group produced higher level of inflammatory cytokines including *iNOS* and *TNF-α,* compared with blank control group. This is consistent with previous study demonstrating that M1-Exo contains RNAs, proteins as well as proinflammatory factors that originated from M1-macrophage, allowing M1-Exo to promote M1 macrophage phenotype shift and create a local immunostimulatory microenvironment [22]. The expression of *iNOS*, *TNF-α, Arg-1* and *CD206* was not significantly changed after either M0-Exo treatment, suggesting naïve macrophage-derived exosomes did not activate macrophage polarization. In contrast, both DTX-M0-Exo and DTX-M1-Exo significantly induced the high expression level of inflammatory cytokines *iNOS* and *TNF-α,* which has similar pattern as observed in LPS/IFN-γ group. However, bothDTX-M0-Exo andDTX-M1-Exo delivery systems achieved differentiation of M0 macrophages to M1 macrophages. Meanwhile, the expression levels of *Arg-1* were down-regulated for DTX-M0-Exo and DTX-M1-Exo groups, while the expression levels of *CD206* were down-regulated for DTX and DTX-M1-Exo groups, as shown in Fig. 4C, D. Meanwhile, DTX also showed slightly lower expression levels of *Arg-1* and *CD206*, and higher expression levels of inflammatory cytokines *iNOS* and *TNF-α* compared with blank control group. Above results validated precious study that DTX modulated TAM polarization to inflammatory M1 phenotype via inhibition of STAT3 pathway [16]. Collectively, the cytokine expression profiles suggested that DTX-M1-Exo exhibited the most significant inhibition of M2 polarization, while induce the polarization of naïve macrophages into inflammatory M1 form in vitro.

### DTX-M1-Exo prolonged M1 polarization after IL-4 restimulation

**Fig. 5:**
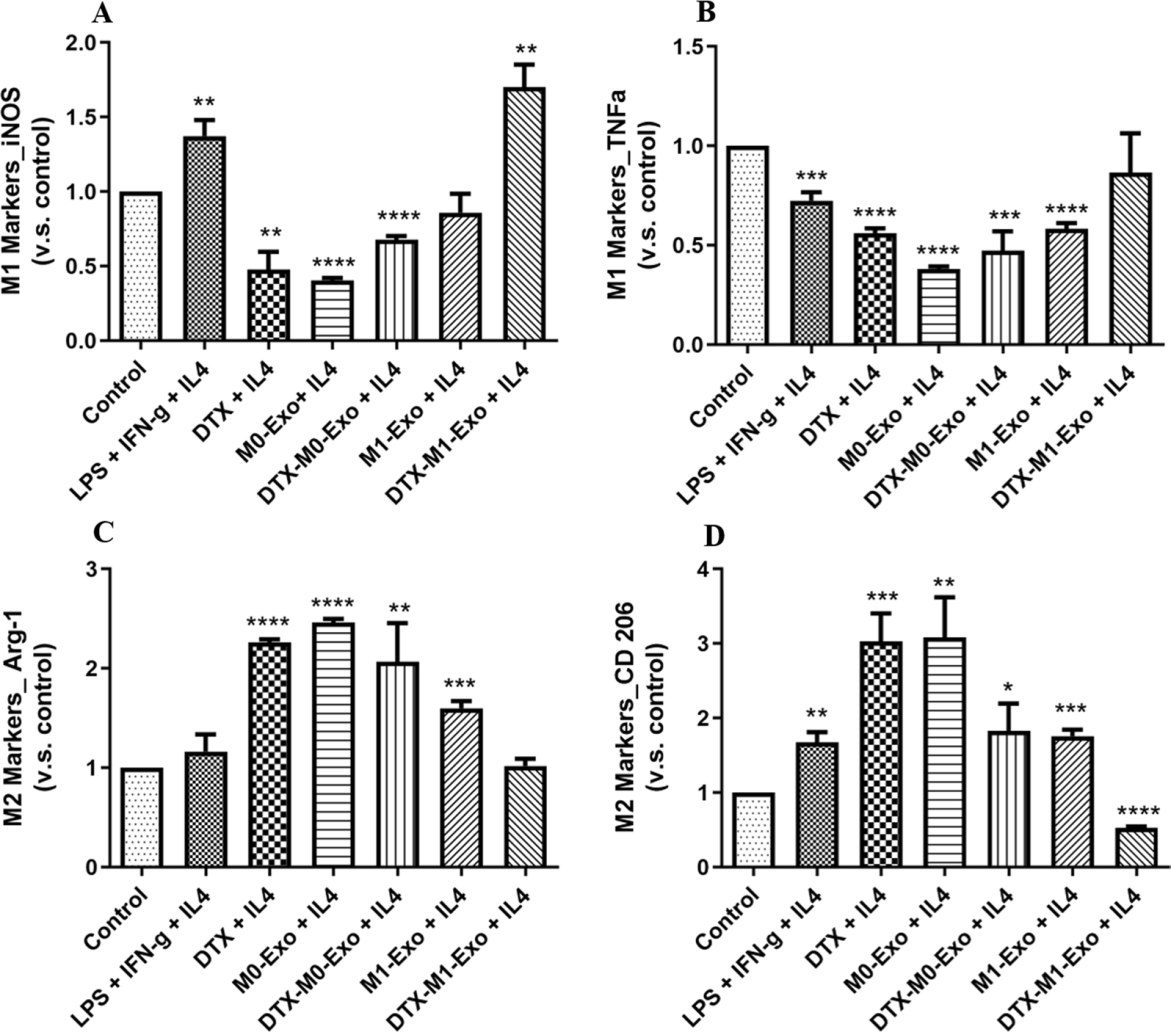
Macrophage repolarization capability of IL-4-induced M2 macrophages, followed by the treatment of LPS + IFN-γ, DTX, M0-Exo, DTX-M0-Exo, M0-Exo, and DTX-M1-Exo, by measuring the expression of M1-macrophage markers (**A**) *iNOS* and (**B**) *TNF-α*, as well as M2-macrophage markers (**C**) *Arg-1* and (**D**) *CD206.* (* *P* < 0.05, ** *P* < 0.01, *** *P* < 0.001, **** *P* < 0.0001 vs. the control group)

Most cancer cells promote M2-like TAMs to generate an immunosuppressive tumor microenvironment and facilitate tumor metastasizes. Therefore, it is challenging to continuously repolarize M2-like TAMs into tumoricidal M1 macrophages and maintain high potency of M1 polarization. To evaluate repolarization capacity of the macrophages treated with DTX-M1-Exo, we pre-treated naïve macrophages with IL-4 to mimic M2 macrophage-dominant tumor microenvironment, and then assessed the cytokine expression profiles of the macrophages after treatments. As shown in Fig. 5, we found that when treat stimulated M2 macrophages with DTX-M1-Exo, they showed high expression of *iNOS* and *TNF-α,* while low expression levels of *Arg-1* and *CD206*. Above results suggested that DTX-M1-Exo treatment induced M1 macrophage polarization with long-lasting potent ability. On the contrary, DTX group and DTX-M0-Exo group failed to increase the expression levels of the M1 markers in were reduced in response to IL-4 stimulation. In the meantime, they increased the expression of the M2 markers, indicating that macrophages treated with either DTX or DTX-M0-Exo were not able to be repolarized to M1 phenotype in tumor microenvironment. Overall, our results showed that DTX-M1-Exo prolonged M1 macrophage polarization even upon M2 macrophage stimulation. We demonstrated that DTX-M1-Exo prevented M1 macrophage repolarization to M2, which can reverse immunosuppressive tumor microenvironment and benefit the cancer therapy.

### DTX-M1-Exo caused mitochondrial dysfunction on naïve macrophages

**Fig. 6:**
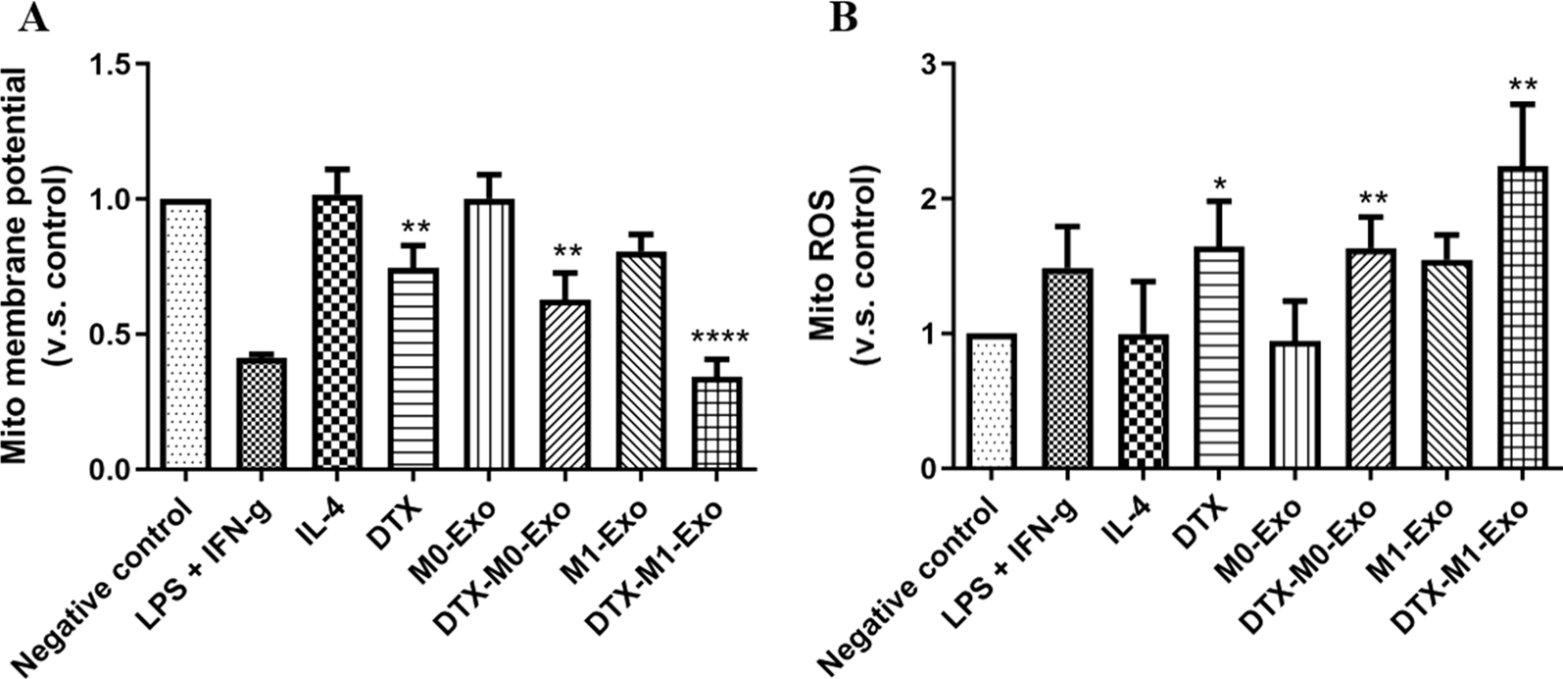
Mitochondria function of macrophages with IL-4, LPS + IFN-γ, DTX, M0-Exo, DTX-M0-Exo, M0-Exo, and DTX-M1-Exo, determined by (**A**) mitochondrial membrane potential changes and (**B**) mitochondrial ROS induction. (* *P* < 0.05, ** *P* < 0.01, **** *P* < 0.0001)

It is known that mitochondrial metabolism is associated with macrophage polarization and function alteration. More recently, studies demonstrated that mitochondria health plays an important role in macrophage repolarization [14, 15]. To further explore the mechanism of DTX-M1-Exo induced M1 macrophage repolarization, we characterized the mitochondrial function by measuring mitochondrial membrane potential changes and mitochondrial reactive oxygen species (mitoROS) production in response to different formulations. Mitochondrial membrane potential is a key indicator of mitochondrial membrane activities during OXPHO [23], while reduction of mitochondrial membrane potential is a sign of mitochondria damage. In this study, the treated macrophages were stained by a mitochondrial membrane potential dye TMRM and analyzed by flow cytometry. As showed in Fig. 6A, DTX-M1-Exo decreased the level of mitochondrial potential to the greatest extent in naïve macrophages compare to other groups, suggesting abnormal mitochondrial function. In addition, loss of mitochondrial membrane potential is linked with accumulation of mitochondrial ROS. We then monitored the levels of mitochondrial ROS using a fluorescent dye MitoSOX and found that DTX-M1-Exo generated the highest level of mitochondrial ROS compared with either DTX group or DTX-M0-Exo group (Fig. 5B). These findings indicated that DTX-M1-Exo inducing mitochondrial dysfunction results a significant contribution to improved M1 polarization capability, which can increase the cancer-killing ability of tumoricidal M1 macrophages.

### M1-Exo-DTX reduced tumor burden and enhanced anti-tumor efficiency in vivo

**Fig. 7:**
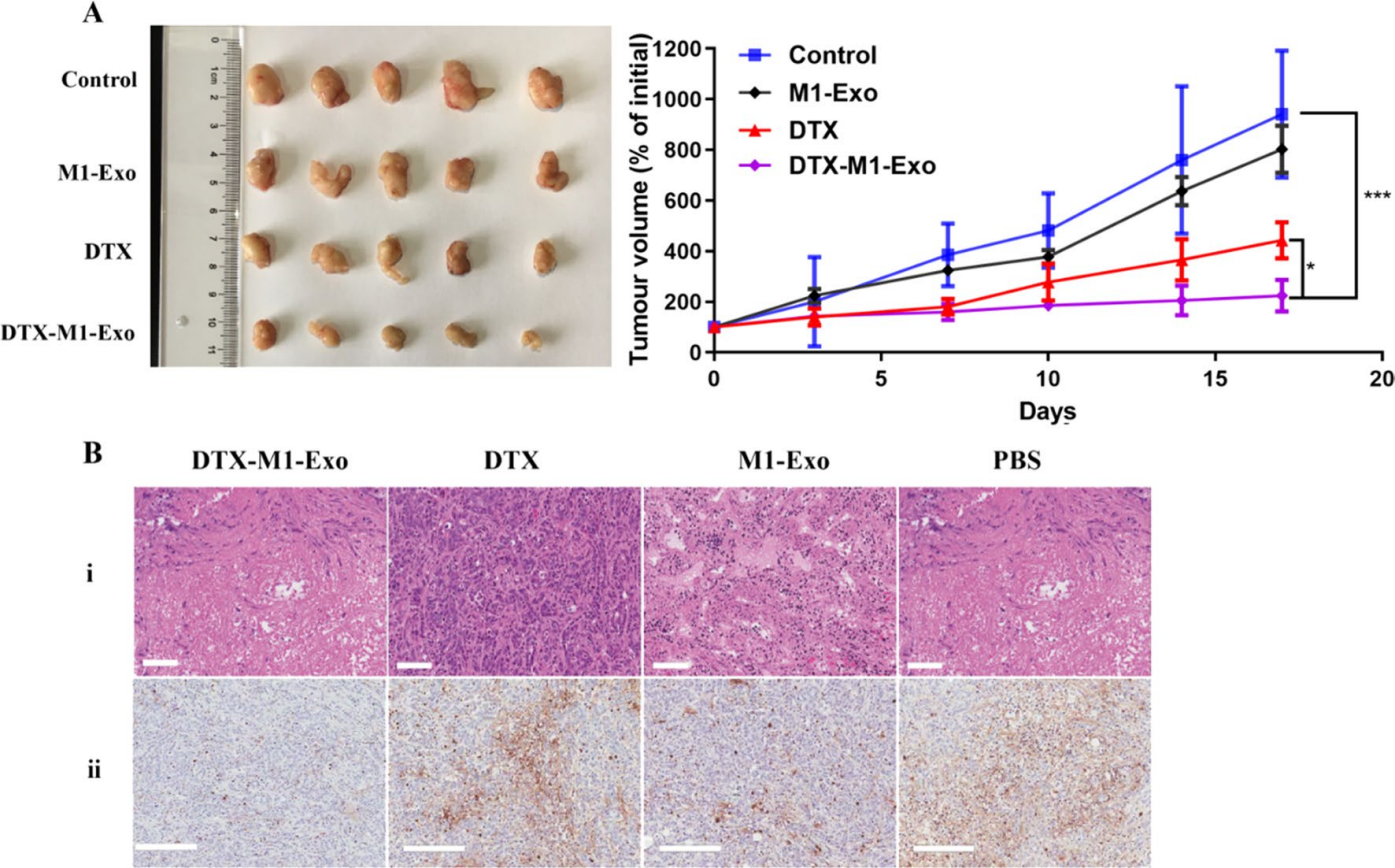
Tumor growth inhibition of breast cancer 4T1in BALB/C mice. Mice were injected i.v. with PBS control, M1-Exo, DTX, and DTX-M1-Exo at concentration of 5 mg/kg DTX: (**A**) Tumor volume changes. (**B**) Representative histological images of slices of 4T1 tumor after final treatment using (i) H&E staining (scale bar = 100 μm) and (ii) Ki67 staining (scale bar = 200 μm)

Above in vitro experiments have shown that DTX-M1-Exo had great potential in maintaining potent tumoricidal M1 phenotype. Hence, it may improve cancer treatment by inducing chemotherapy-based cancer killing and modulating tumor immune microenvironment. To verify the in vivo anti-tumor efficiency of DTX-M1-Exo, BALB/C mice bearing 4T1 murine mammary carcinoma were established and injected with PBS control, M1-Exo, DTX, or DTX-M1-Exo. Both DTX-M1-Exo and DTX groups showed certain suppression of tumor growth when compared to administration of PBS (Fig. 7A). The treatment of DTX-M1-Exo showed the highest inhibition of tumor growth among all the groups (p < 0.05). The reduction in tumor size in vivo agrees with the in vitro cytotoxicity study. In addition, M1-Exo group showed a slightly higher level of suppression tumor growth as compared to PBS group, which may be associated with macrophage-mediated tumoricidal activity of M1-Exo.

Inhibition of tumor growth was further validated by staining a nuclear protein Ki67 which is associated with tumor cell proliferation (Fig. 7B) In Fig. 7Bii, the treatment of DTX-M1-Exo significantly decreased the number of Ki67-positive cells (brown staining) in the tumors, compared with other groups, indicating the reduction of tumor burden. This is consistent with the results of tumor volume changes. Meanwhile, histological images of the H&E-stained tumor sections excised from the different treatment groups showed that DTX-M1-Exo induced dramatic cell death in the tumors compared to that of either M1-Exo or free DTX (Fig. 7Bi). These results suggest that DTX-M1-Exo can significantly improve the therapeutic efficacy of DTX at both cellular and in vivo levels.

### M1-Exo-DTX increased macrophage infiltration in tumor

**Fig. 8:**
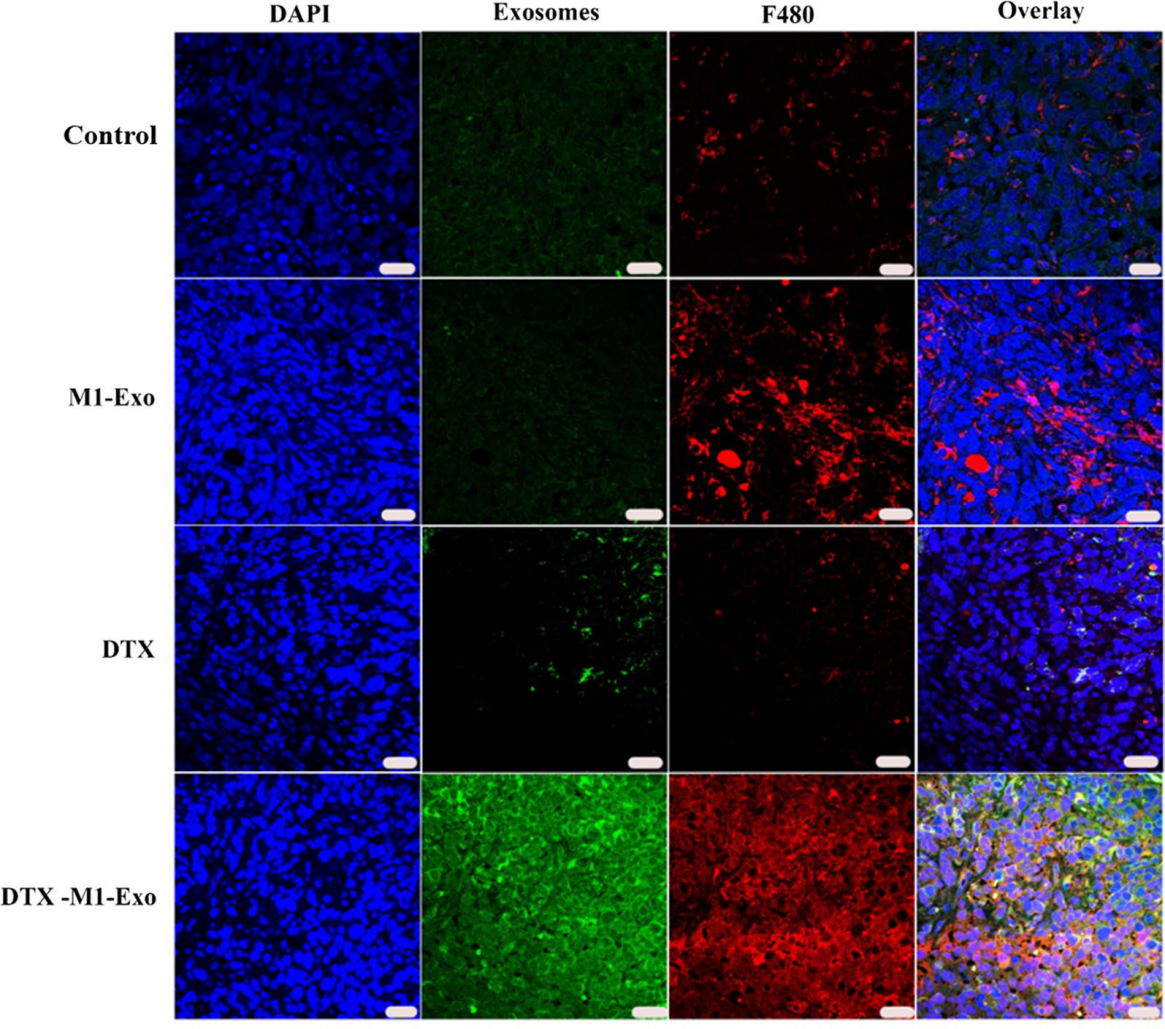
The distribution of exosome formulations and macrophages in vivo. Representative fluorescent images showing nucleus (blue), PKH67-labelled exosomes (green), F480-labelled macrophages (red), and merged channels (from left to right). The scale bar denotes 20 μm

To evaluate macrophage activation and intratumoral distribution of M1-Exo-DTX, the tumor tissues were fixed, sectioned, stained and imaged using confocal microscopy. We found that significantly higher amount of DTX-M1-Exo and M1-Exo were accumulated in the tumor region, compared with other groups (Fig. 8). This tumor homing behavior of M1-Exo is consistent with the results reported in the literature [24]. Our data demonstrated that M1-Exo is an ideal candidate for chemotherapeutic drug delivery. Furthermore, DTX-M1-Exo treated tumor also showed higher level of F480 expression, suggesting that DTX-M1-Exo plays a positive role in macrophage infiltration and activation in the tumor tissues. To investigate macrophage programming, we then stained tumor tissues with M1 marker CD86 and M2 marker CD163. As shown in Additional file 1: Figs. S1 and S2, DTX-M1-Exo treated tumor had lowest level of CD163-positive macrophages compared to other groups, and in contrast, DTX-M1-Exo treated tumor had highest level of CD86-positive macrophages, which support our previous finding that DTX-M1-Exo enabled potent tumoricidal M1 polarization. This is confirmed by accumulation of CD86-positve macrophages observed in the DTX-M1-Exo group (Additional file 1: Fig. S2). These findings indicated that M1-Exo, especially, DTX-M1-Exo caused mitochondrial dysfunction, leading to inhibition of M2 phenotype polarization and prolonged the immunotherapeutic effects of M1 macrophages.

### In vivo safety assessment

**Fig. 9:**
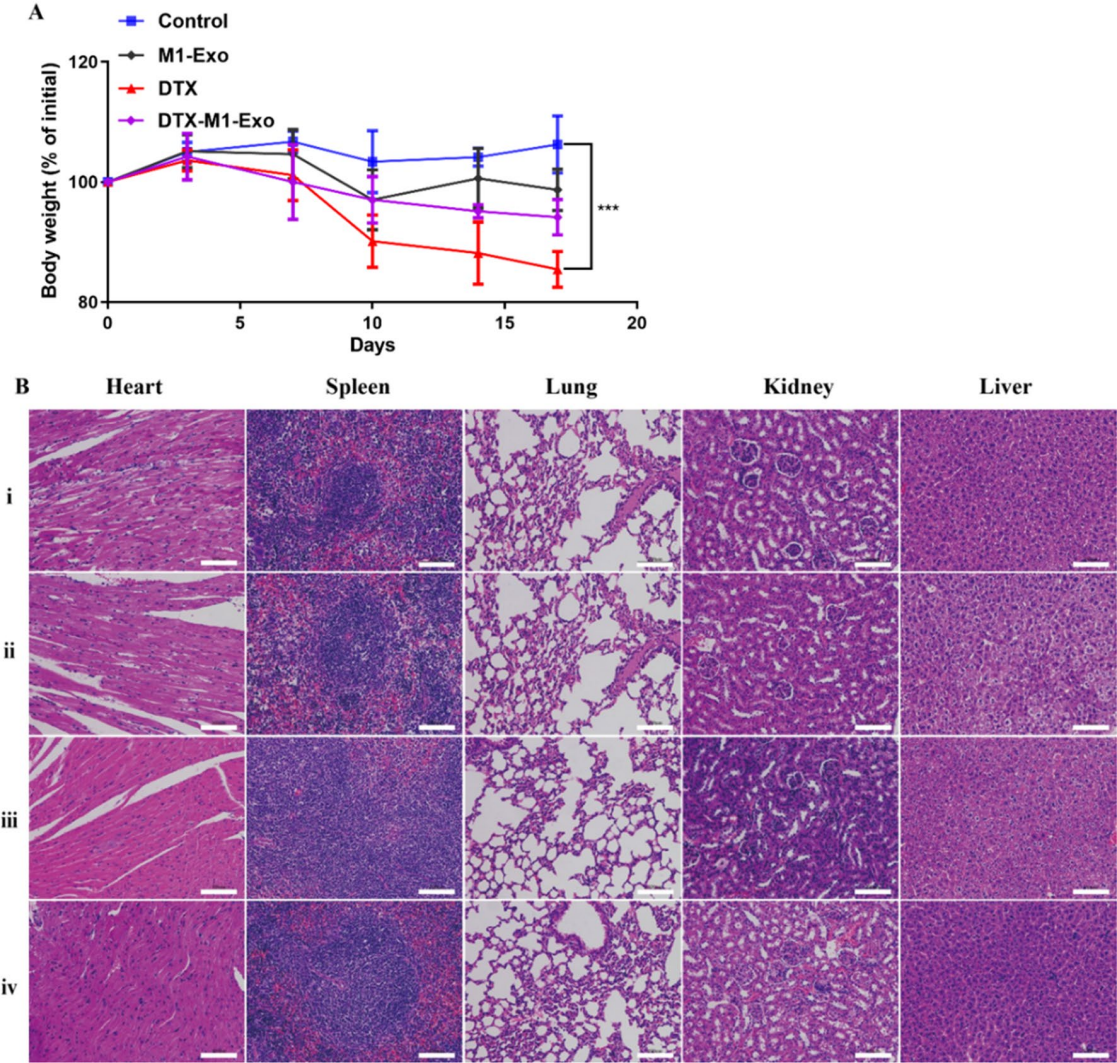
In vivo biosafety assessment. **A** Body weight changes. **B** Representative histological images of H&E-stained slices of each organ two days after treatment of (i) control, (ii) DTX, (iii) M1-Exo, and (iv) DTX-M1-Exo. The scale bar is 100 μm. (n = 3)

The in vivo biosafety profiles of DTX-M1-Exo were systematically investigated and presented in Fig. 9. After intravenous administration of the exosome formulations, both DTX-M1-Exo and M1-Exo showed minimal toxic effects in the mice, where they exhibit no significant weight loss during the therapeutic regime compared to the PBS control group (Fig. 9A). In contrast, the DTX group displayed statistically significant weight loss. Next, the pathological examination of major organs, including the liver, heart, lung and kidney was performed using H&E staining. As shown is Fig. 9B, no obvious morphological changes or lesions in the harvested organs were observed in DTX-M1-Exo, M1-Exo, or PBS control treatment group. While DTX group showed hydropic degeneration or necrosis in major organs, suggesting this chemotherapeutic agent caused some tissue injury. The results indicated that DTX-M1-Exo has potent anticancer efficacy for breast cancer together with good biocompatibility.

## Conclusion

M1-macrophage-derived exosome delivery platform, DTX-M1-Exo, was successfully prepared and characterized. We demonstrated that DTX-M1-Exo promoted polarization of naïve macrophages to M1 phenotype, at the same time, and maintained M1 form upon M2 stimulation through modulating mitochondrial function. In vivo tumor regression study showed that DTX-M1-Exo significantly improved the anti-cancer therapeutic efficacy with minimal side effect. Therefore, we conclude that this M1-Exo-based delivery system provide a novel strategy for macrophage programming by therapeutically targeting mitochondria function, which has great potential for cancer management.

## Supplementary Information

**Additional file 1: Figure S1.** Tumor slice stained with DAPI and M2 marker CD163. **Figure S2** Tumor slice stained with DAPI and M1 marker CD86.
