# Supplementary material for: Docetaxel-loaded M1 macrophage-derived exosomes for a safe and efficient chemoimmunotherapy of breast cancer
Source: J Nanobiotechnology. 2022 Aug 2;20:359. doi: 10.1186/s12951-022-01526-2 (PMC9344780; doi:10.1186/s12951-022-01526-2)
Supplement: Supplementary file 1 — Additional file 1: Figure S1. Tumor slice stained with DAPI and M2 marker CD163. Figure S2 Tumor slice stained with DAPI and M1 marker CD86. [file 12951_2022_1526_MOESM1_ESM.docx]

**Docetaxel-loaded M1 macrophage-derived exosomes for a safe and efficient** **chemoimmunotherapy of breast cancer**

Yongmei Zhao^1^, Yuanlin Zheng^1^, Yan Zhu^1^, Hongyun Li^1^, Hongyan Zhu^1*^, Tianqing Liu^2*^

^1^ School of Pharmacy, Nantong University, Nantong, China

^2^ NICM Health Research Institute, Western Sydney University, Westmead, Australia


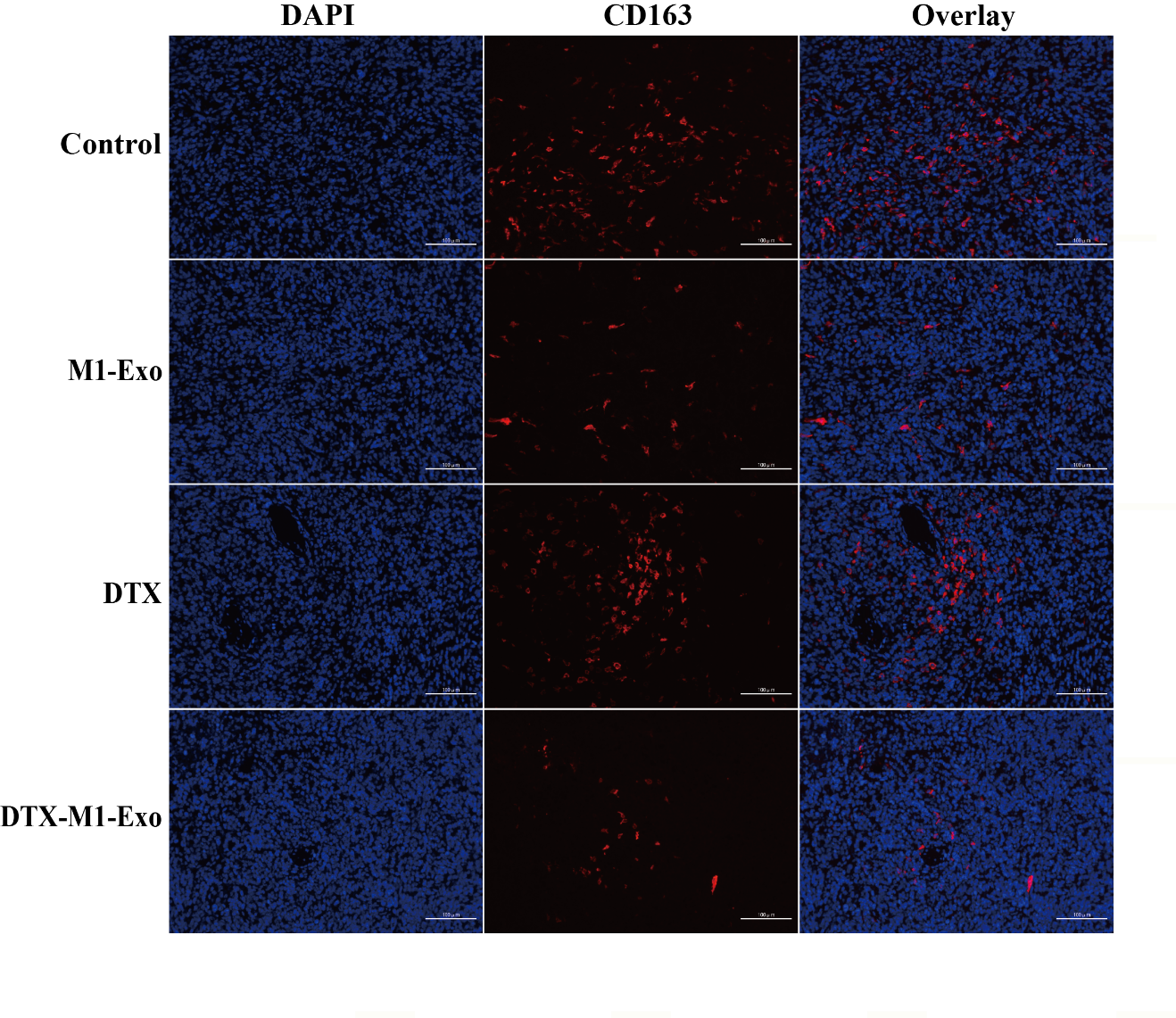


**Figure S1** Tumor slice stained with DAPI and M2 marker CD163.


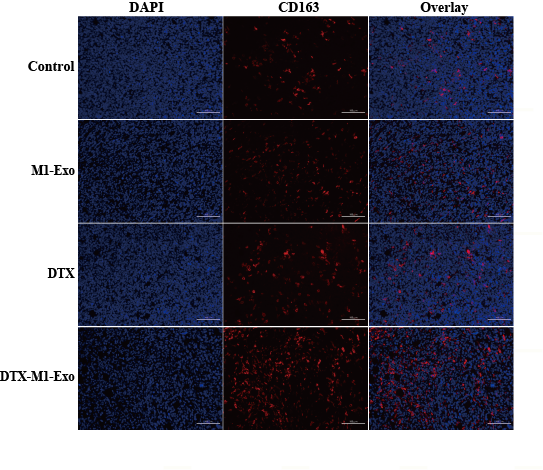


**Figure S2** Tumor slice stained with DAPI and M1 marker CD86.
